# Real-world pharmacovigilance analysis of galsulfase: a study based on the FDA adverse event reporting system (FAERS) database

**DOI:** 10.3389/fphar.2024.1420126

**Published:** 2024-08-05

**Authors:** Shangze Li, Runcheng Huang, Yuanyuan Meng, Yijia Liu, Jiao Qian, Junjie Zou, Jun Yang

**Affiliations:** ^1^ Department of Orthopedics, The Second Affiliated Hospital (Changzheng Hospital), Naval Medical University, Shanghai, China; ^2^ Department of Endocrinology, The Second Affiliated Hospital (Changzheng Hospital), Naval Medical University, Shanghai, China; ^3^ Department of Traditional Chinese Medicine, The First Affiliated Hospital (Changhai Hospital), Naval Medical University, Shanghai, China; ^4^ Department of Ultrasound, The Second Affiliated Hospital (Changzheng Hospital), Naval Medical University, Shanghai, China; ^5^ Department of Pharmacy, The First Affiliated Hospital (Changhai Hospital), Naval Medical University, Shanghai, China

**Keywords:** galsulfase, Naglazyme, mucopolysaccharidosis VI (MPS VI), pharmacovigilance, adverse events, FDA adverse event reporting system (FAERS)

## Abstract

**Background:**

Associated with enzyme deficiencies causing glycosaminoglycans (GAGs) accumulation, mucopolysaccharidosis type VI (MPS VI) is lysosomal storage disorder. In the treatment of MPS VI, galsulfase (Naglazyme) is commonly used as an enzyme replacement therapy (ERT). There remains a need for comprehensive real-world data on its safety and associated adverse events (AEs).

**Objective:**

An analysis of the FDA Adverse Event Reporting System (FAERS) database will be conducted to identify potential risks and adverse reactions associated with galsulfase in real-life settings.

**Methods:**

The FAERS database was used to extract data from Q2 2005 to Q4 2023. A total of 20,281,876 reports were analyzed after duplicate elimination, with 3,195 AE reports related to galsulfase identified. The association between galsulfase and AEs was investigated by utilizing four algorithms: reporting odds ratio (ROR), proportional reporting ratio (PRR), Bayesian confidence propagation neural network (BCPNN), and multi-item gamma Poisson shrinker (MGPS). The analysis focused on the timing of onset, signs of AEs, and clinical significance.

**Results:**

Twenty seven organ systems were involved, and significant system organ classes (SOCs) included respiratory, thoracic and mediastinal disorders, and infections and infestations. At the PT level, 72 PTs corresponding to 15 SOCs were identified, with some AEs not previously mentioned in the product label. AEs associated with galsulfase had a median onset time of 1,471 days, with over half of the cases occurred within the first 5 years of treatment initiation.

**Conclusion:**

This investigation delivers an exhaustive and indicative assessment of galsulfase’s safety profile, grounded in authentic, real-world evidence. The findings emphasis the importance of continuous safety surveillance and the emergence of new AEs. The identification of previously unreported urologic adverse events, such as glomerulonephritis membranous and nephritic syndrome, warrants further investigation. The study emphasizes the need for enhanced pharmacovigilance to ensure patient safety and the effectiveness of galsulfase treatment.

## 1 Introduction

The mucopolysaccharidoses (MPSs) are a collection of lysosomal storage disorders resulting from deficiencies in enzymes essential for glycosaminoglycans (GAGs) metabolism (Brunelli et al., 2021; Li et al., 2024). GAGs, being a diverse range of extracellular heteropolysaccharides, serve various roles in human physiology (Chin and Fuller, 2022). The progressive and systemic symptoms commonly observed in early childhood are attributed to the accumulation of GAGs in various tissues (Harmatz et al., 2014). The progressively deteriorating manifestations encompass skeletal, cardiovascular and respiratory, hematological, visual, auditory, and cognitive impairments (Sestito et al., 2022; Al Kaissi et al., 2023; Hwang-Wong et al., 2023; Miller et al., 2023; Ratiani and Leventaki, 2023). The multi-organ impact of this condition not only impedes daily activities but also disrupts social interactions, emotional well-being, and academic performance in children (Li et al., 2024).

There are three primary methods currently in use of treating MPS VI: transplantation of hematopoietic stem cells, enzyme replacement therapy, and gene-based treatments (Penon-Portmann et al., 2023). Galsulfase, known commercially as Naglazyme, is widely used enzyme replacement medication for MPS VI. GAGs are catabolized more efficiently by galsulfase when it is incorporated into lysosomes, thereby increasing their degradation (Valayannopoulos et al., 2010).

Prior investigations have indicated early initiation of galsulfase therapy could potentially prevent or mitigate the advancement of certain disease manifestations (Sohn et al., 2012; Harmatz et al., 2014; 2017; Lin et al., 2016; Gomes et al., 2019). However, previous studies mostly focused on the efficacy of drugs, and the data were mostly derived from clinical trials rather than real-world studies, so there was a lack of systematic studies on drug adverse events (AEs). Skinner et al. searched for articles about enzyme replacement therapies and found that only 7% mentioned AEs (Skinner et al., 2018). To improve the correct understanding of AEs can effectively define the scope of treatment and the way of using of drugs, and timely put forward reasonable and scientific modification opinions on the current package inserts.

Therefore, searching for the AEs of galsulfase in the real-world is essential. The FAERS database serves as a comprehensive repository for post-marketing surveillance, housing authentic AE reports sourced from a multitude of contributors (Zou et al., 2024). The database is readily accessible for public download on the FDA’s official website (Wang et al., 2024). Our main objective is using four algorithms to search for possible risks linked to galsulfase, and expect to provide guidance for clinical application and further enrich the application scenarios and adverse reactions of drugs.

## 2 Methods

### 2.1 Data source and collection

**FIGURE 1:**
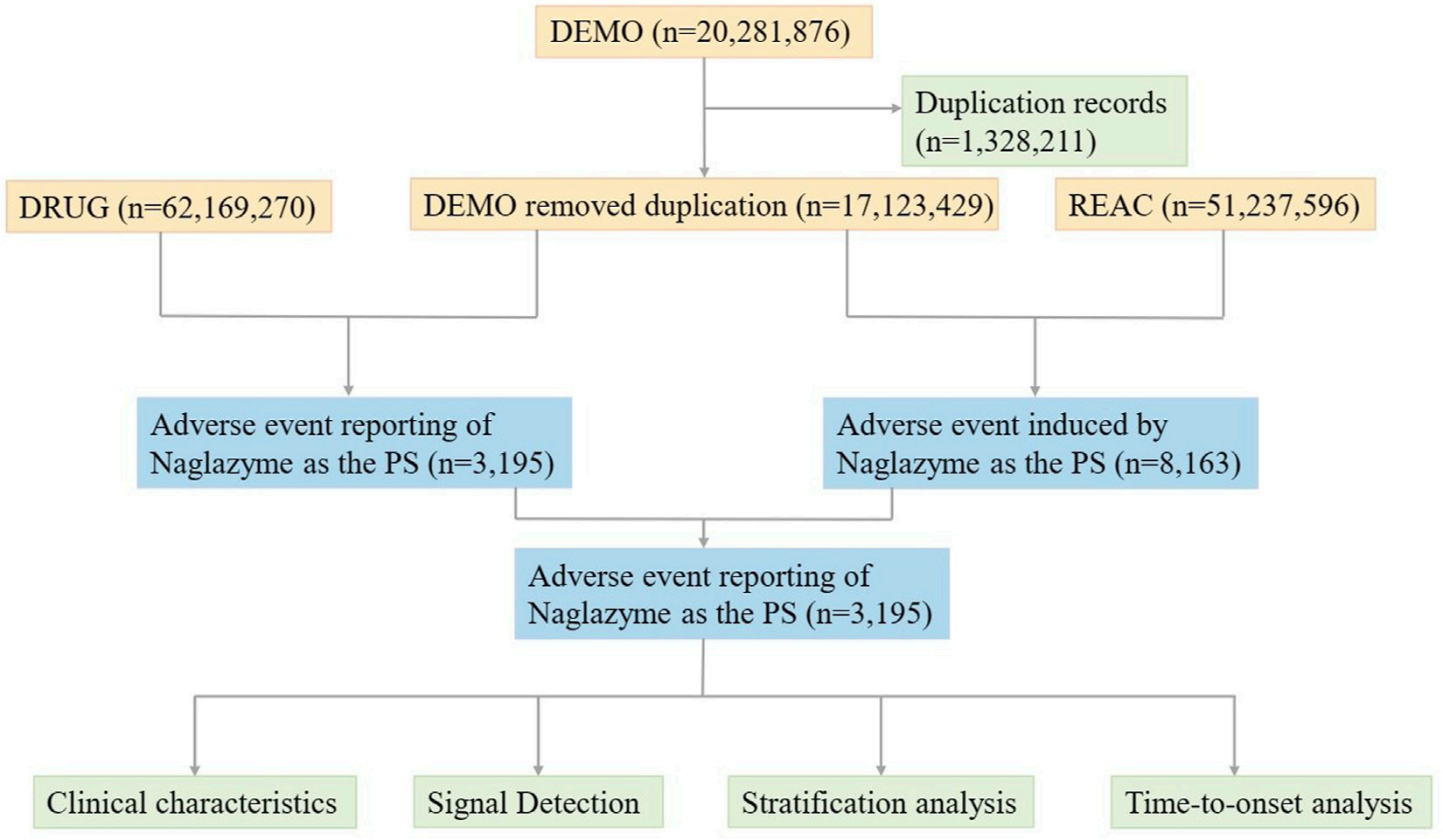
The process of selecting galsulfase-associated AEs from FAERS database.

Utilizing the FAERS database, pharmacovigilance data on galsulfase in the post-marketing phase from Q2 2005 to Q4 2023 was gathered. Designed to assist the FDA in monitoring the safety profiles of approved drugs and therapeutic biologic products post-approval, FAERS is founded on Individual Case Safety Reports (ICSRs: E2B) as issued by the International Council for Harmonisation of Technical Requirements for Pharmaceuticals for Human Use (ICH). Adverse events (AEs) are systematically classified in line with the Medical Dictionary for Regulatory Activities (MedDRA). FAERS comprises seven distinct datasets including demographic and administrative details (DEMO), information on adverse drug reactions (REAC), patient outcomes (OUTC), drug specifics (DRUG), drug therapy start and end dates (THER), details regarding reporting sources (RPSR), and indications for use or diagnosis (INDI). The database collates reports of AEs, product quality concerns, and medication errors impacting patient safety. In order to identify and remove duplicate reports, priority was given to the most recent FDA_DT when CASEID values matched. Conversely, when both CASEID and FDA_DT coincided, a PRIMARYID with a higher value was selected (Shu et al., 2022). During the study period, the FAERS database recorded a total of 20,281,876 reports related to galsulfase. Following the removal of duplicate entries, 17,123,429 case reports identified galsulfase as the main suspect drug, associating it with 3,195 adverse events (Figure 1). All reports on galsulfase were systematically categorized based on System Organ Class (SOC) and Preferred Term (PT) levels. The codes for drug roles in events (ROLE_COD) were classified as primary suspect (PS), secondary suspect (SS), concomitant (C), or interacting (I). Furthermore, both the generic name (galsulfase) and brand name (naglazyme) were identified as target drugs in the DRUG file.

### 2.2 Statistical analysis

The relationship between galsulfase and AEs was evaluated using statistical algorithms such as the Reporting Odds Ratio (ROR), Proportional Reporting Ratio (PRR), Bayesian Confidence Propagation Neural Network (BCPNN), and Multi-Item Gamma Poisson Shrinker (MGPS). These assessments were based on disproportionality analysis. Detailed information on the equations and criteria for these algorithms can be found in (Table 1). Our study analyzed data on AE signals that met the specific criteria of each algorithm. Signals indicating novel AEs were identified as any significant adverse event not previously documented in the product information (Full Prescribing Information, Revised: 12/2019). The onset time was defined as the duration between the occurrence of the AE (EVENT_DT) and the initiation of galsulfase treatment (START_DT). Reports with data entry errors, such as EVENT_DT preceding START_DT or containing incorrect dates, were excluded from the analysis. The onset time was described using the median and interquartile range (IQR). Statistical software programs R 4.3.3, Navicat Premium 15, and Microsoft Excel 2019 were utilized for data processing and statistical computations.

**TABLE 1:** The specific formulas of the four algorithms.

Algorithms	Equation	Criteria
ROR	ROR = ad/bc	Lower limit of 95%CI > 1, a ≥ 3
	95%CI = eln^(ROR)±1.96(1/a+1/b+1/c+1/d)^0.5^	
PRR	PRR = [a (c + d)]/[c (a + b)]	PRR ≥ 2, χ^2^ ≥ 4, a ≥ 3
	χ^2^ = [(ad-bc)^^^2](a + b + c + d)/[(a + b) (c + d) (a + c) (b + d)]	
BCPNN	IC = log_2_a (a + b + c + d)/[(a + c) (a + b)]	IC025 > 0
	95%CI = E (IC) ± 2 [V(IC)]^^^0.5	
MGPS	EBGM = a (a + b + c + d)/[(a + c) (a + b)]	EBGM05 > 2
	95%CI = eln^(EBGM)±1.96(1/a+1/b+1/c+1/d)^0.5^	

Notes: Equation: a, number of reports containing both the target drugs and the target adverse drug reactions; b, number of reports containing other adverse drug reactions of the target drugs; c, number of reports containing the target adverse drug reactions of other drugs; d, number of reports containing other drugs and other adverse drug reactions. Abbreviations: ROR, reporting odds ratio; PRR, proportional reporting ratio; BCPNN, Bayesian Confidence Propagation Neural Network; MGPS, multi-item gamma Poisson Shrinker; 95% CI, 95% confidence interval; χ2, chi-squared; IC, information component; IC025, the lower limit of the 95% CI of the IC; E, the IC expectations; V, the variance of IC; EBGM, empirical Bayesian geometric mean; EBGM05, empirical Bayesian geometric mean lower 95% CI for the posterior distribution.

## 3 Results

### 3.1 General characteristics

**FIGURE 2:**
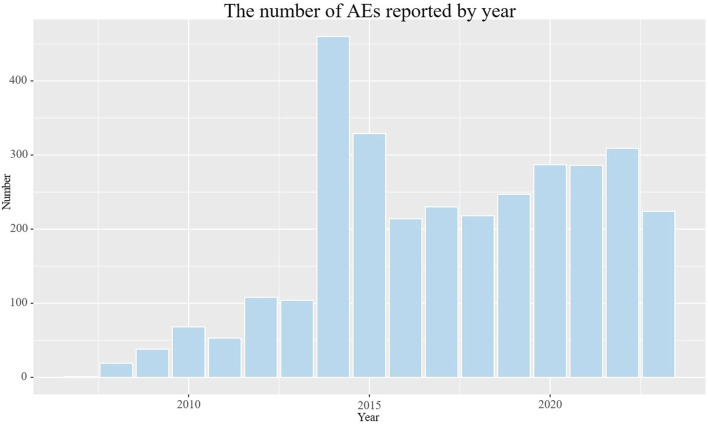
The number of AEs reported by year.

The detailed clinical characteristics of studies regarding galsulfase can be found in (Table 2). In terms of gender, females (45.4%) experienced a higher incidence of adverse events compared to males (37.5%). In age distribution, a greater proportion was observed among patients under 18 years old (37.9%), surpassing both those over 65 years old and those aged between 18 and 65 years. The most commonly reported indication was MPS VI (78.0%), followed by unclassified mucopolysaccharidosis (3.4%). The highest number of AEs (40.8%) was reported by the United States, with Brazil (25.5%), Colombia (7.1%), the United Kingdom (4.2%), and Germany (2.8%) following. Serious consequences encompass fatalities, life-threatening situations, hospital stays, impairments, and other severe outcomes. In order to establish the distribution of each type, we computed the percentage of each category in relation to the overall serious outcome submissions. Among the serious outcomes, hospitalization emerged as the predominant type, constituting 31.7%. The remaining serious outcomes were documented in 934 instances, accounting for 25.7%, whereas fatalities were recorded in 281 cases, equating to 7.7%. Excluding reports from unknown sources, consumers and physicians were the primary reporters of adverse events, accounting for 74.1% and 12.6% respectively. The number of reported adverse events exhibited a gradual increase over the initial 8 years, followed by a period of stabilization (Figure 2). The peak year for reported AEs was 2014 (14.4%), followed by 2015 (15.3%), 2022 (9.7%), 2020 (9.0%), 2021 (9.0%), and 2019 (7.7%), respectively.

**TABLE 2:** Clinical characteristics of reports with galsulfase from the FAERS database (from the second quarter of 2005 to the fourth quarter of December 2023).

Characteristics	Case Number, n	Case proportion, %
Number of events	3,195	
Gender		
Female	1,451	45.4
Male	1,198	37.5
Unknown	546	17.1
Age		
<18	1,210	37.9
18∼65	545	17.1
>65	7	0.2
Unknown	1,433	44.9
Weight		
<50 kg	1,525	47.7
50∼100 kg	199	6.2
>100 kg	8	0.3
Unknown	1,463	45.8
Reported person		
Consumer	2,369	74.1
Physician	403	12.6
Health professional	167	5.2
Other health-professional	147	4.6
Pharmacist	8	0.3
Registered nurse	3	0.1
Unknown	98	3.1
Reported countries (top five)		
America	1,289	40.3
Brazil	814	25.5
Colombia	226	7.1
United Kingdom	135	4.2
German	90	2.8
Serious outcomes	n = 3,640	
Hospitalization (HO)	1,155	31.7
Other serious outcomes (OT)	934	25.7
Death (DE)	281	7.7
Life-threatening (LT)	87	2.4
Disability (DS)	51	1.4
Congenital anomaly (CA)	8	0.2
Required intervention (RI)	1	0.0
Unknown	1,123	30.9
Indications(top five)	n = 2,938	
Mucopolysaccharicosis VI	2,291	78.0
Product used for unknown indication	516	17.6
Mucopolysaccharidosis	100	3.4
Mucopolysaccharisosis IV	22	0.7
Lipidosis	3	0.0

### 3.2 Signal detection

#### 3.2.1 Signals of system organ class (SOC)

The galsulfase-related adverse events showed varying signal strengths at the level of SOC as outlined in (Table 3). Analysis of the collected data revealed that AEs linked to galsulfase affected a total of 27 organ systems. Noteworthy SOCs meeting the specified criteria included disorders of the respiratory, thoracic, and mediastinal systems [n = 1,219, ROR (95%CI) = 3.56 (3.35–3.78)], incidences of infections and infestations [n = 1,136, ROR (95%CI) = 2.92 (2.74–3.11)], occurrences related to surgical and medical procedures [n = 289, ROR (95%CI) = 2.75 (2.44–3.09)], problems with the ear and labyrinth [n = 87, ROR (95%CI) = 2.46 (1.99–3.04)], as well as conditions involving congenital, familial, and genetic factors [n = 66, ROR (95%CI) = 2.58 (2.02–3.28)]. Additionally, disorders of the nervous system [n = 865, ROR (95%CI) = 1.28 (1.19–1.37)], cardiac issues [n = 401, ROR (95%CI) = 1.92 (1.73–2.12)], and vascular problems [n = 205, ROR (95%CI) = 1.18 (1.02–1.35)] were identified as significant SOCs meeting at least one of the specified criteria.

**TABLE 3:** Signal strength of reports of galsulfase at the SOC level in FAERS database.

SOC	N	ROR (95%Cl)	PRR (χ^2^)	IC (IC025)	EBGM(EBGM05)
Respiratory, thoracic and mediastinal disorders	1,219	3.56 (3.35 – 3.78)^a^	3.18 (1907.87)^a^	1.67 (0)	3.18 (3.02)^a^
Infections and infestations	1,136	2.92 (2.74 – 3.11)^a^	2.65 (1,236.24)^a^	1.41 (−0.26)	2.65 (2.52)^a^
Nervous system disorders	865	1.28 (1.19 – 1.37)^a^	1.25 (45.92)	0.32 (−1.35)	1.25 (1.17)
Cardiac disorders	401	1.92 (1.73 – 2.12)^a^	1.87 (166.88)	0.9 (−0.76)	1.87 (1.72)
Surgical and medical procedures	289	2.75 (2.44 – 3.09)^a^	2.68 (309.54)^a^	1.42 (−0.24)	2.68 (2.43)^a^
Vascular disorders	205	1.18 (1.02 – 1.35)^a^	1.17 (5.27)	0.23 (−1.44)	1.17 (1.04)
Ear and labyrinth disorders	87	2.46 (1.99 – 3.04)^a^	2.45 (74.85)^a^	1.29 (−0.37)	2.45 (2.05)^a^
Congenital, familial and genetic disorders	66	2.58 (2.02 – 3.28)^a^	2.56 (63.07)^a^	1.36 (−0.31)	2.56 (2.09)^a^

^a^ Indicates statistically significant signals in algorithms. Notes: The SOCs that met at least one of the algorithm screening criterions are listed. Abbreviations: SOC, system organ class; n, the number of reports; ROR, reporting odds ratio; 95%CI, 95% confidence interval; PRR, proportional reporting ratio; χ2, chi-squared; IC, information component; IC025, the lower limit of 95%CI of the IC; EBGM, empirical Bayesian geometric mean; EBGM05, the lower limit of 95%CI of EBGM.

#### 3.2.2 Signals of preferred terms (PTs)

**FIGURE 3:**
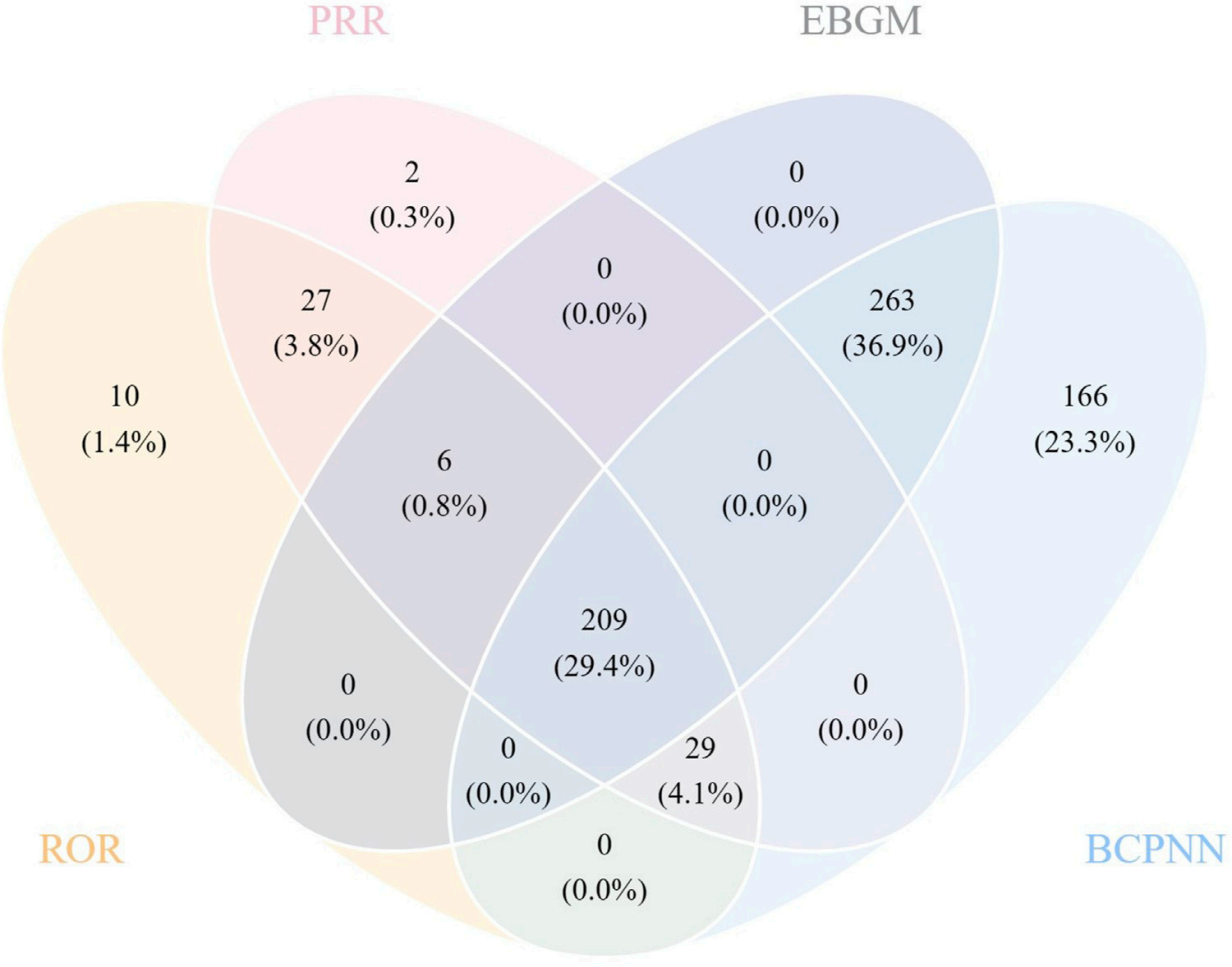
Venn diagram for the screening of all PTs based on the results of the four algorithms.

All four algorithms together detected 209 instances of PTs induced by galsulfase, impacting 21 System Organ Classes (SOCs), as illustrated in (Supplementary Table S1). The detailed screening process is depicted in (Figure 3). (Table 4) Offers a summarized list of reported post-marketing surveillance AEs that occurred with a minimum frequency of 11 times. This table encompasses 72 PTs, which are associated with 15 different SOCs. After comparing the package insert, 33 PTs were found to have the same drug instructions, which included fever, hernia, breathing difficulties, stuffy nose, infection of the upper respiratory tract, clouding of the cornea, hernia near the belly button, an adverse reaction to infusion, pain at the catheter site, flu, infection of the respiratory tract, infection of the lower respiratory tract, ear infection, streptococcal throat infection, coughing, breathing difficulties, pain in the throat and mouth, high blood pressure in the lungs, bronchial spasms, cough with mucus, rapid breathing, breathing failure, runny nose, respiratory issue, sore throat, blockage in the airway, decreased oxygen levels, high body temperature, and hearing loss.

**TABLE 4:** Signal strength of reports of galsulfase at the PT level in FAERS database.

SOC	PT	n	ROR (95%Cl)
General disorders and administration site conditions	Pyrexia	332	7.44 (6.67 – 8.31)
	Secretion discharge	24	15.72 (10.53 – 23.48)
	Catheter site pain	16	51.25 (31.32 – 83.87)
	Complication associated with device	15	4.24 (2.56 – 7.04)
	Hernia	14	5.57 (3.3 – 9.41)
	Catheter site swelling	12	104.46 (59.02 – 184.88)
Infections and infestations	Pneumonia	273	6.22 (5.51 – 7.01)
	Influenza	75	5.44 (4.33 – 6.83)
	Ear infection	56	16.69 (12.83 – 21.71)
	Device related infection	43	18.59 (13.77 – 25.1)
	Upper respiratory tract infection	41	6.76 (4.97 – 9.19)
	Viral infection	30	7.15 (5 – 10.24)
	Respiratory tract infection	29	8.86 (6.15 – 12.76)
	Lower respiratory tract infection	18	3.26 (2.05 – 5.18)
	Otitis media	13	29.08 (16.86 – 50.16)
	Pharyngitis streptococcal	12	8.57 (4.86 – 15.09)
Respiratory, thoracic and mediastinal disorders	Cough	178	4.96 (4.28 – 5.76)
	Respiratory failure	54	5.6 (4.28 – 7.32)
	Rhinorrhoea	54	6.46 (4.94 – 8.45)
	Sleep apnoea syndrome	49	18.39 (13.88 – 24.36)
	Respiratory disorder	47	11.98 (8.99 – 15.97)
	Oropharyngeal pain	40	3.21 (2.35 – 4.38)
	Respiratory distress	38	10.42 (7.58 – 14.34)
	Nasal congestion	37	4.94 (3.57 – 6.82)
	Pulmonary hypertension	28	9.85 (6.79 – 14.27)
	Bronchospasm	27	14.31 (9.8 – 20.88)
	Productive cough	26	4.37 (2.98 – 6.43)
	Tachypnoea	23	13.45 (8.93 – 20.26)
	Obstructive airways disorder	21	14.09 (9.18 – 21.64)
	Respiratory arrest	15	3.86 (2.33 – 6.41)
	Apnoea	15	14.33 (8.63 – 23.79)
	Acute respiratory failure	14	5.75 (3.4 – 9.71)
	Increased bronchial secretion	12	43.95 (24.9 – 77.57)
Nervous system disorders	Spinal cord compression	133	265.14 (222.61 – 315.79)
	Seizure	78	3.45 (2.76 – 4.32)
	Cervical cord compression	54	1,381.17 (1,028.08 – 1855.53)
	Carpal tunnel syndrome	44	23.28 (17.3 – 31.33)
	Hydrocephalus	41	57.6 (42.32 – 78.4)
	Intracranial pressure increased	19	26.1 (16.62 – 40.98)
	Myelopathy	11	37.92 (20.95 – 68.62)
Investigations	Oxygen saturation decreased	100	14.5 (11.9 – 17.67)
	Body temperature increased	17	6.14 (3.81 – 9.88)
Musculoskeletal and connective tissue disorders	Cervical spinal stenosis	58	250.75 (192.7 – 326.28)
	Spinal stenosis	19	15.75 (10.04 – 24.72)
	Scoliosis	16	18.58 (11.37 – 30.36)
	Kyphosis	14	56.53 (33.38 – 95.71)
Vascular disorders	Cyanosis	47	22.95 (17.22 – 30.59)
	Poor venous access	23	17.27 (11.46 – 26.02)
	Pallor	19	5.16 (3.29 – 8.1)
Cardiac disorders	Tachycardia	46	3.96 (2.96 – 5.29)
	Cardio-respiratory arrest	36	6.39 (4.6 – 8.86)
	Mitral valve incompetence	31	20.81 (14.62 – 29.63)
	Aortic valve incompetence	17	30.48 (18.92 – 49.12)
	Cardiac valve disease	15	19.34 (11.65 – 32.13)
	Cardiomegaly	13	7.59 (4.41 – 13.09)
	Mitral valve disease	12	50.08 (28.36 – 88.42)
Eye disorders	Corneal opacity	31	188.14 (131.54 – 269.1)
	Blindness	22	4.16 (2.74 – 6.33)
Gastrointestinal disorders	Umbilical hernia	28	44.82 (30.88 – 65.04)
	Inguinal hernia	25	29.96 (20.21 – 44.4)
Injury, poisoning and procedural complications	Infusion related reaction	34	4.17 (2.98 – 5.84)
	Head injury	20	4.82 (3.1 – 7.47)
Surgical and medical procedures	Tracheostomy	26	143.21 (97.03 – 211.38)
	Corneal transplant	19	207.86 (131.54 – 328.45)
	Hip surgery	16	25.12 (15.37 – 41.06)
	Spinal operation	15	10.81 (6.51 – 17.95)
	Ear tube insertion	13	359.98 (205.75 – 629.81)
	Spinal decompression	13	253.77 (145.7 – 442.01)
	Knee operation	12	10.28 (5.83 – 18.11)
Ear and labyrinth disorders	Deafness	19	5.59 (3.56 – 8.77)
Congenital, familial and genetic disorders	Developmental hip dysplasia	13	87.21 (50.43 – 150.82)
Product issues	Device occlusion	13	7.9 (4.58 – 13.61)

Abbreviations: SOC, system organ class; PT, preferred term; n, the number of reports; ROR, reporting odds ratio; 95%CI, 95% confidence interval. Notes: The PTs with n ≥ 11 and met the four algorithm screening criterions are listed.

Significantly, our efforts in data mining revealed numerous noteworthy adverse events that were not specifically cited in the galsulfase product label. These included 20 adverse event reports included pneumonia, seizure, cervical spinal stenosis, ear infection, sleep apnoea syndrome, cyanosis, tachycardia, carpal tunnel syndrome, device related infection, hydrocephalus, cardio-respiratory arrest, mitral valve incompetence, viral infection, tracheostomy, inguinal hernia, secretion discharge, poor venous access, blindness, obstructive airways disorder. Some PTs were discovered with increased signal intensity, including ear tube insertion [n = 13, ROR (95%CI) = 359.98 (205.75–629.81)], spinal decompression [n = 13, ROR (95%CI) = 253.77 (145.7–442.01)], cervical spinal stenosis [n = 58, ROR (95%CI) = 250.75 (192.7–326.28)], corneal transplant [n = 19, ROR (95%CI) = 207.86 (131.54–328.45)], tracheostomy [n = 26, ROR (95%CI) = 143.21 (97.03–211.38)], catheter site swelling [n = 12, ROR (95%CI) = 104.46 (59.02–184.88)]. Our research has revealed extra adverse events that enhance the overall comprehension of the safety characteristics of galsulfase.

#### 3.2.3 Onset time of AEs

**FIGURE 4:**
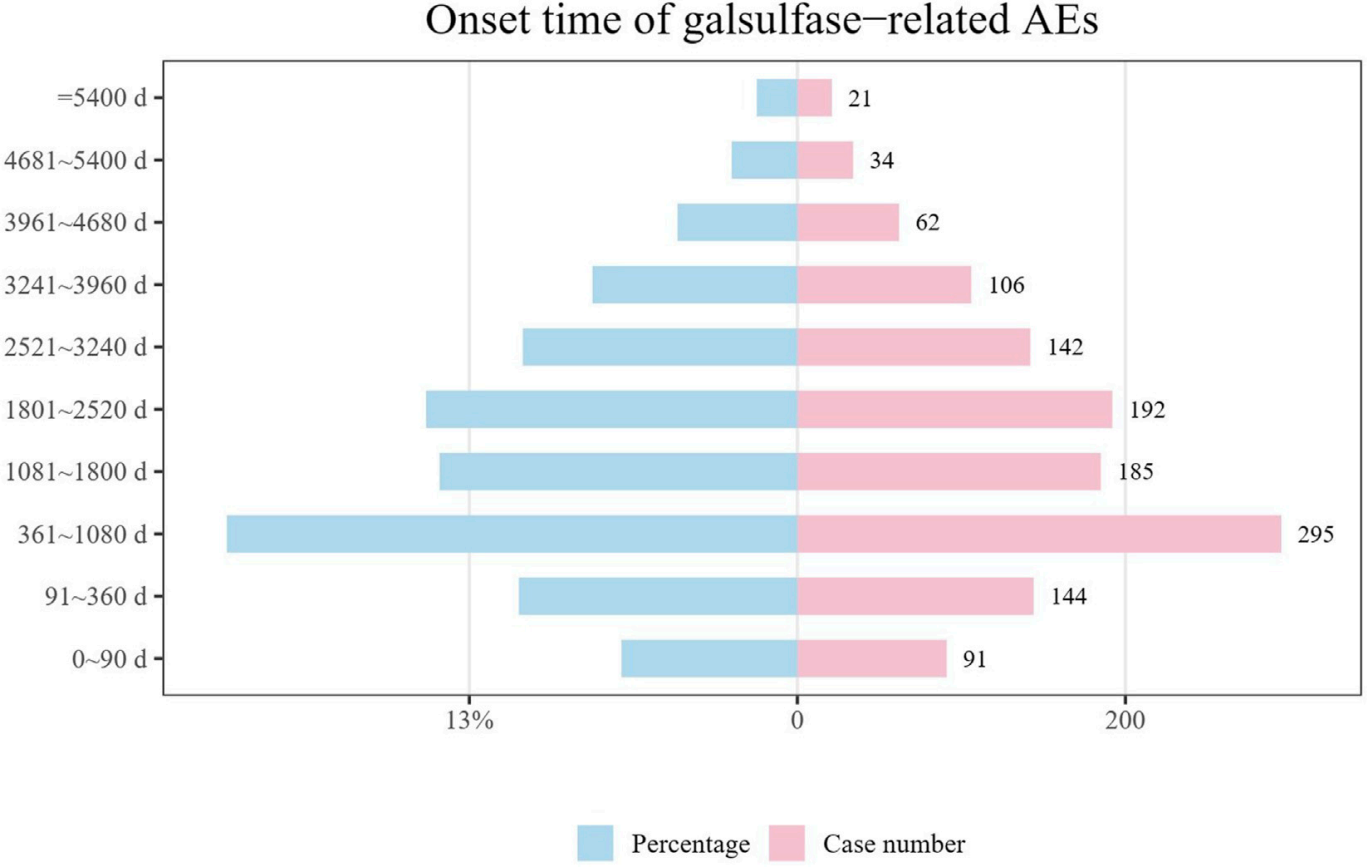
Onset time of galsulfase-related AEs.

Data on the initiation periods for adverse events linked to galsulfase were collected from the FAERS repository. Upon removal of inaccurate accounts, a sum of 1,272 records containing documented initiation periods remained. The median initiation period stood at 1,471 days, accompanied by IQR spanning from 523 to 2,706.25 days. As illustrated in (Figure 4), the findings suggest that the onset of adverse events related to galsulfase can be widespread, potentially spanning over a decade. Nevertheless, more than half of these cases (n = 715, 56.20%) occurred within the initial 5 years following the commencement of galsulfase treatment. The incidence of AES in 1 year (n = 235, 18.47%), 3 years (n = 295, 23.19%), 5 years (n = 185, 14.54%), 7 years (n = 192, 15.09%), 9 years (n = 142, 11.16%), 11 years (n = 106, 8.33%) decreased gradually, which reflected that patients may have a better long-term effect of medication.

## 4 Discussion

MPS VI, characterized as a rare genetic disease with a low incidence rate, affects a limited population of patients but multiple systems result from the build-up of GAGs in connective tissues (Valayannopoulos et al., 2010). This phenomenon is caused by a lack of the enzyme N-acetylgalactosamine 4-sulfatase (Tebani et al., 2019; Jones et al., 2020). In an autosomal recessive manner, this genetic disorder is inherited, arising from mutations in the ARSB gene on chromosome 5q13-q14. Over 130 different mutations have been found, primarily consisting of missense mutations (Valayannopoulos et al., 2010; Ghaffari et al., 2022). Galsulfase has been demonstrated improvement in walking and stair-climbing abilities (Sohn et al., 2012). In a phase IV, multinational, open-label study with two dosage levels conducted in infants in 2014, it was shown that urinary GAG levels turn down after galsulfase treatment (Harmatz et al., 2014). A case series study in 2016 provided evidence that galsulfase decreased levels of urinary GAG and enhanced clinical functions over a long-term period (Lin et al., 2016).

Although galsulfase has been widely used in the treatment of MPS VI, its safety and adverse reactions still need to be further studied to ensure the safety and effectiveness of patients (Lampe et al., 2019; D’Avanzo et al., 2021). Previous studies on galsulfase have primarily been clinical trials or retrospective studies, with a focus on case series, and the majority of these studies have enrolled no more than 55 patients (Harmatz et al., 2005; Brands et al., 2013; Miller et al., 2023). However, the present study stands out due to its significantly larger overall sample size (n = 3,195), which is derived from real-world data, volunteered by applicants from various occupational sources, thereby enhancing its reliability. This large sample size is crucial for validating previously reported AEs associated with galsulfase and for discovering potential new AEs. The utilization of real-world data allows for a more comprehensive understanding of the safety profile of galsulfase in a diverse and representative patient population, beyond the limitations of smaller, more controlled study settings. Therefore, the current study offers a significant advancement in the field of MPS VI research, contributing to a deeper understanding of the drug’s safety and potential risks.

By utilizing the four algorithms mentioned in (Table 1), we ranked the signal values of the collected AEs (Sakaeda et al., 2013). After analyzing the galsulfase related data, we found that at the SOC level, although there were 8 SOCs with significant statistical signals according to at least 1 algorithm: respiratory, thoracic and mediastinal disorders [n = 1,219, ROR (95%CI) = 3.56 (3.35–3.78)], infections and infestations [n = 1,136, ROR (95%CI) = 2.92 (2.74–3.11)], nervous system disorders [n = 865, ROR (95%CI) = 1.28 (1.19–1.37)], cardiac disorders [n = 401, ROR (95%CI) = 1.92 (1.73–2.12)], surgical and medical procedures [n = 289, ROR (95%CI) = 2.75 (2.44–3.09)], vascular disorders [n = 205, ROR (95%CI) = 1.18 (1.02–1.35)], ear and labyrinth disorders [n = 87, ROR (95%CI) = 2.46 (1.99–3.04)], congenital, familial and genetic disorders [n = 66, ROR (95%CI) = 2.58 (2.02–3.28)] (Table 3), according to recent advances in clinical research on MPS VI, the above-mentioned statistically significant SOC were all associated with the progression of MPS VI disease (Harmatz et al., 2005; Hoang et al., 2019; Inci et al., 2021; Al Kaissi et al., 2023; Miller et al., 2023; Ratiani and Leventaki, 2023). To our astonishment, general disorders and administration site conditions contributed the most frequent AEs (n = 1,225), but it didn’t meet the screening criteria of any of the algorithms shown.

At the PT levels, we evaluated all post-marketing surveillance AEs that fulfilled the screening criteria of all four algorithms and summarized the corresponding SOC levels (statistics for all PTs are provided in (Supplementary Table S2). All PTs listed in (Supplementary Table S2) passed the Bonferroni correction, as detailed in (Supplementary Table S3). It was interesting to find that, the most numerous SOCs were respiratory, thoracic and mediastinal disorders (n = 795), infections and infestations (n = 689), and general disorders and administration site conditions (n = 465). Additionally, taking into account the potential progression of MPS VI disease resulting in symptoms affecting the respiratory, musculoskeletal, cardiovascular, nervous, and other systems, we manually eliminated the related System Organ Classes (SOCs) (Valayannopoulos et al., 2010; Garcia et al., 2021). In conclusion, the most frequently occurring SOC was general disorders and administration site conditions. Our research findings align with previous relevant studies (Horovitz et al., 2021; Inci et al., 2021; Kowalski et al., 2022).

Associated with the general disorders and conditions of the administration site, statistically significant PTs included pyrexia (n = 332), discharge of secretions (n = 24), pain at catheter site (n = 16), complication associated with the device (n = 15), hernia (n = 14), swelling at catheter site (n = 12), extravasation at infusion site (n = 8), erythema at catheter site (n = 8), extravasation (n = 7), hyperthermia (n = 5), inflammation at catheter site (n = 4), extravasation at catheter site (n = 4), bruising at catheter site (n = 4), pain from hernia (n = 3), loss of leg control (n = 3), adhesion (n = 3), granuloma (n = 3). Additionally, attention was also drawn to infusion-related reactions (n = 34) and occlusion of the device (n = 13). The majority of these PTs are infusion-associated events which can be technicality avoided (Kim et al., 2008; D’Avanzo et al., 2021). Based on the results of the above analysis, we make a recommendation to change the route of administration from intravenous infusion to automated pump administration, which is to reduce the number and frequency of invasive procedures on the one hand and more stable drug administration on the other. Alternatively, gene therapy is expected to completely solve MPS VI and fundamentally improve the survival treatment and life expectancy of patients (Fachel et al., 2022).

Drawing upon our preceding scientific inquiries, we manually sifted through and excluded PTs that exhibited a correlation with the progression of MPS VI disease. In the process, we discovered two previously undocumented urologic AEs: glomerulonephritis membranous [n = 4, ROR (95%CI) = 25.84 (9.68–69)] and nephritic syndrome [n = 3, ROR (95%CI) = 69.76 (22.35–17.69)]. This revelation offers a fresh perspective in our comprehension of the potential side effects associated with the administration of the drug. Although the precise underlying mechanism remains elusive, we hypothesize that these newly identified AEs may be attributed to the direct or indirect toxic effects of galsulfase on renal cells. Alternatively, they could be a consequence of the immune response elicited by galsulfase, as suggested by previous studies (White et al., 2008a; White et al., 2008b). To further elucidate the specific pathogenesis of these AEs, additional biological and biomolecular investigations are imperative. In light of these novel findings, we strongly recommend the conduct of more comprehensive epidemiologic studies and clinical trials. These endeavors will aid in validating the potential association between glomerulonephritis membranous, nephritic syndrome, and galsulfase, while also assessing the severity and incidence of these AEs. Such an approach will provide crucial insights into the safety profile of this drug and inform future therapeutic strategies.

Through a subgroup analysis of all available data, stratified by gender, we observed that gender did not exert a statistically significant influence on the categorization or frequency of AEs. This finding is depicted in (Supplementary Figure S1), which provides a visual representation of the gender-specific distribution of AEs. Nonetheless, in conducting a subgroup analysis of reporting occupation, we identified a potential bias. Notably, a disproportionate majority of 74.15% (n = 3,195) of the individuals reporting AEs were consumers. This imbalance in the reporting population may introduce confounding variables and influence the interpretation of the data. Therefore, (Supplementary Figure S2) highlights this occupational bias, emphasizing the need for cautious interpretation and further investigation to mitigate its potential impact on the overall analysis.

It is crucial to acknowledge that the FAERS database encompasses a diverse array of data sources, with consumers constituting a significant proportion of the reporting population. Consequently, this presents a range of challenges, including potential delays in reporting, instances of missing reports, and inconsistencies in the quality of submissions. These factors, in turn, may introduce biases that must be carefully considered when interpreting the findings derived from this database. Given the complexity of these circumstances and the potential biases involved, it is imperative to exercise caution when interpreting the results of our analyses. While the FAERS database provides valuable insights into AE signals associated with galsulfase, it is essential to recognize its limitations in pharmacovigilance research. Therefore, it is necessary to complement our findings with additional evaluations conducted through rigorous clinical studies. Despite these limitations, our thorough examination of AE signals associated with galsulfase, as well as the discovery of previously unrecognized signals, serves as a solid foundation for future clinical inquiries on this drug. By conducting further clinical studies, we aim to validate our findings and gain a deeper understanding of the safety profile of galsulfase, ultimately contributing to the improvement of patient care and the advancement of medical science.

## 5 Conclusion

After reviewing 20,281,876 entries in the FAERS database between the second quarter of 2005 and the fourth quarter of 2023, and removing any duplicate entries, a total of 3,195 adverse event reports associated with galsulfase were discovered. A pharmacovigilance analysis was employed to uncover the AE signals associated with galsulfase. The onset time, manifestation of AEs, and concomitant medications were examined. The infusion-related symptoms that were frequently reported aligned with the information provided in the galsulfase label, which helps to further corroborate the safety profile. Additionally, new clinically significant AEs were identified: membranous glomerulonephritis and nephritic syndrome. This research underscores the necessity for enhanced safety monitoring to decrease the occurrence of AEs linked to galsulfase and to ensure patient safety.

## Data Availability

The original contributions presented in the study are included in the article/Supplementary Material, further inquiries can be directed to the corresponding authors.
