# Supplementary figures and images for: Real-world pharmacovigilance analysis of galsulfase: a study based on the FDA adverse event reporting system (FAERS) database
Source: Front Pharmacol. 2024 Aug 5;15:1420126. doi: 10.3389/fphar.2024.1420126 (PMC11330839; doi:10.3389/fphar.2024.1420126)

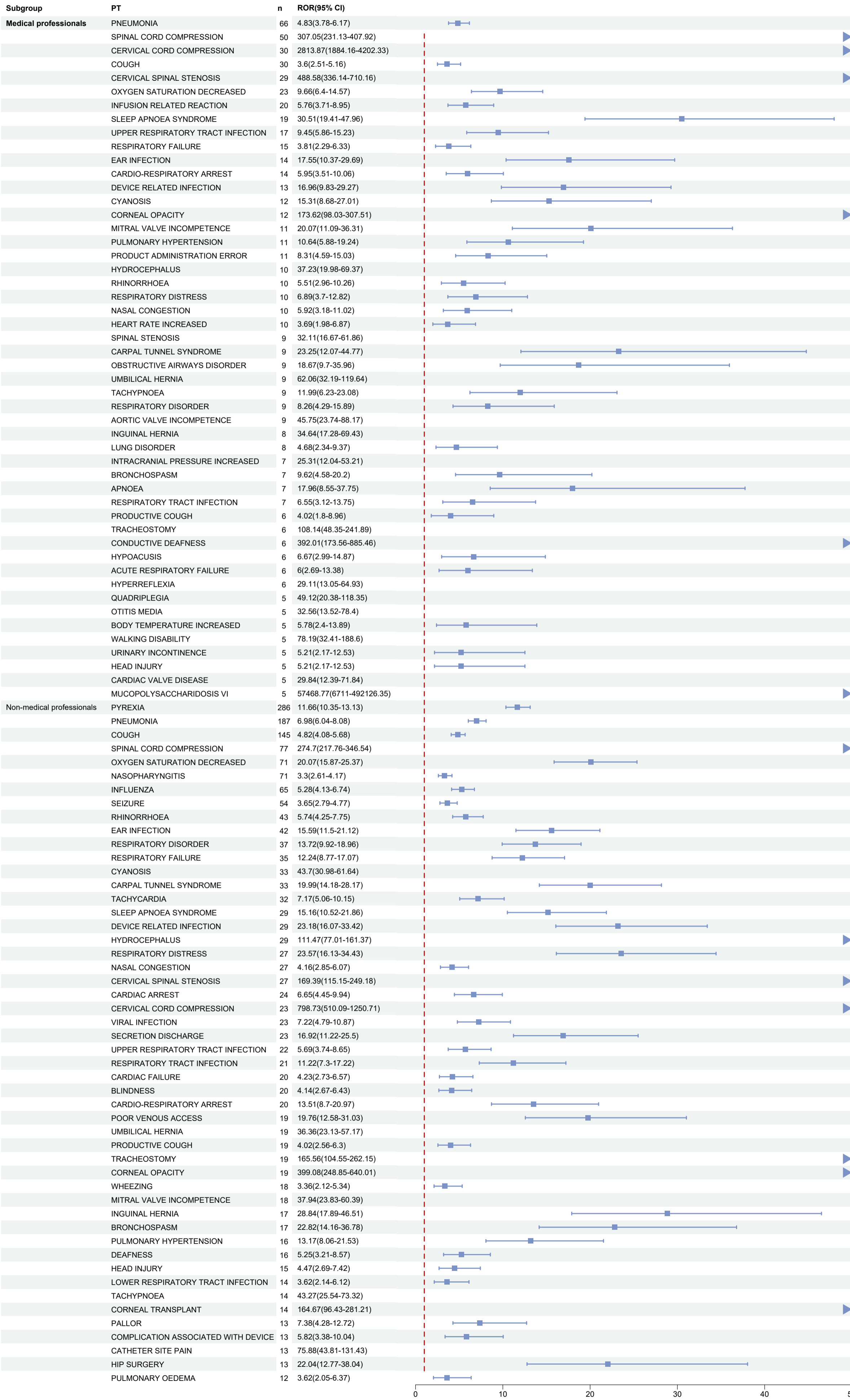

Supplement: Supplementary file 3 [file Image2.pdf]

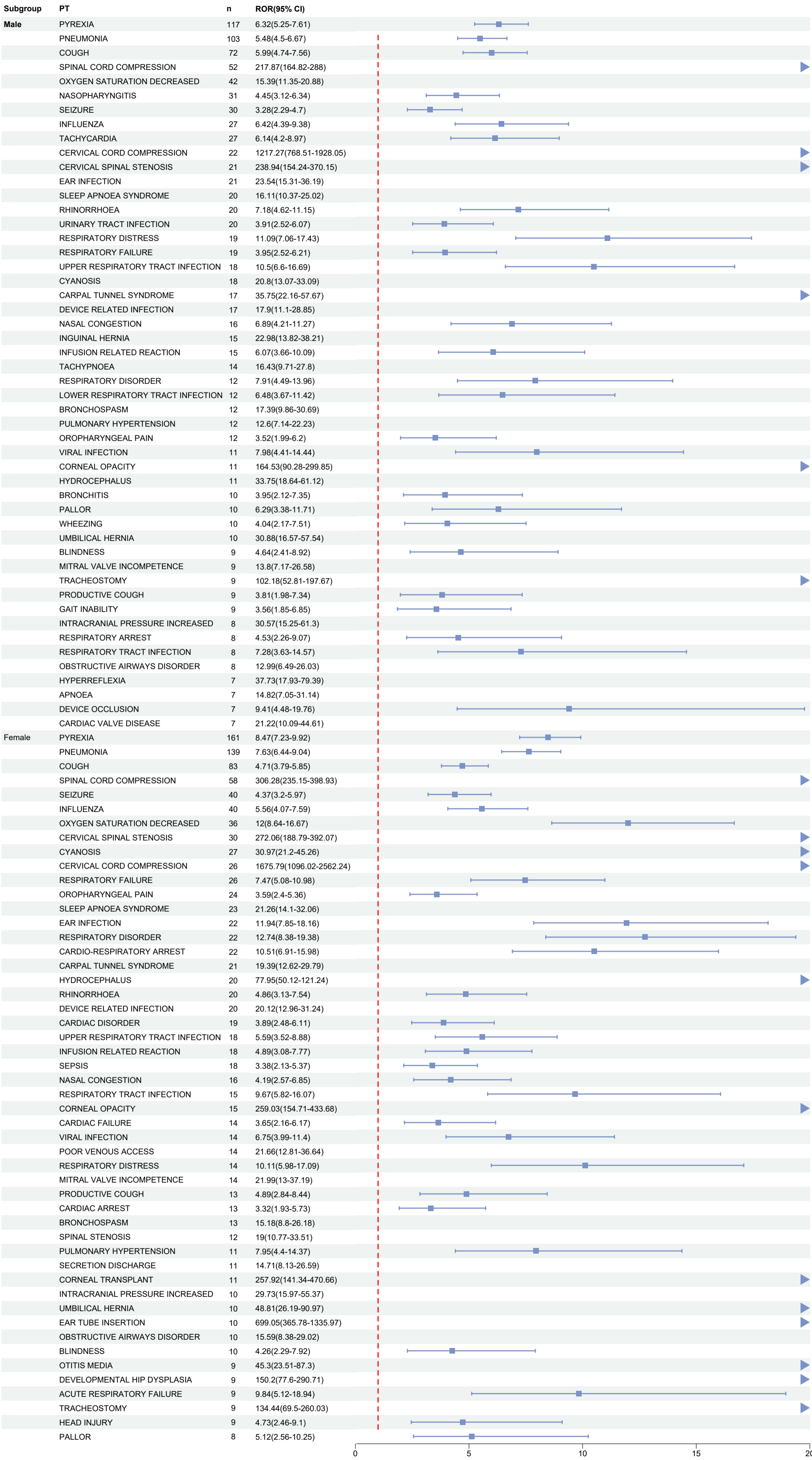

Supplement: Supplementary file 5 [file Image1.pdf]
